# Neighbourhood Socioeconomic Disadvantage and Severe Maternal Morbidity: Secondary Analysis of a Prospective Cohort

**DOI:** 10.1111/1471-0528.70024

**Published:** 2025-09-30

**Authors:** T. Caroline Bank, Janet Catov, Jiqiang Wu, Lynn M. Yee, David M. Haas, Rebecca McNeil, Jessica Pippen, Hyagriv N. Simhan, Uma Reddy, Robert M. Silver, Lisa Levine, George Saade, Judith Chung, Courtney D. Lynch, William A. Grobman, Kartik K. Venkatesh

**Affiliations:** ^1^ Department of Obstetrics and Gynecology The Ohio State University Columbus Ohio USA; ^2^ Department of Obstetrics and Gynecology University of Pittsburgh Pittsburgh Pennsylvania USA; ^3^ Department of Obstetrics and Gynecology Northwestern University Chicago Illinois USA; ^4^ Department of Obstetrics and Gynecology Indiana University Indianapolis Indiana USA; ^5^ RTI International Durham North Carolina USA; ^6^ Department of Obstetrics and Gynecology Case Western Reserve University Cleveland Ohio USA; ^7^ Department of Obstetrics and Gynecology Columbia University New York New York USA; ^8^ Department of Obstetrics and Gynecology University of Utah Salt Lake City Utah USA; ^9^ Department of Obstetrics and Gynecology University of Pennsylvania Philadelphia Pennsylvania USA; ^10^ Department of Obstetrics and Gynecology Eastern Virginia Medical College Norfolk Virginia USA; ^11^ Department of Obstetrics and Gynecology University of California Irvine Orange California USA; ^12^ Department of Obstetrics and Gynecology Brown University Providence Rhode Island USA

**Keywords:** area deprivation index, pregnancy, severe maternal morbidity, socioeconomic disadvantage

## Abstract

**Objective:**

To examine whether neighbourhood socioeconomic disadvantage, as measured by the Area Deprivation Index (ADI) in early pregnancy, was associated with severe maternal morbidity (SMM) at delivery hospitalisation.

**Design:**

A prospective multi‐site observational cohort.

**Setting:**

A secondary analysis of the Nulliparous Pregnancy Outcomes Study: Monitoring Mothers‐To‐Be study (nuMoM2b) across eight United States (US) sites from 2010 to 2013.

**Study Design:**

Participant residential address in the first trimester was geocoded at the US census‐tract level to calculate the ADI, a standardised metric of neighbourhood socioeconomic disadvantage. We used modified Poisson regression with robust error variance and adjusted for individual‐level covariates: age, pre‐pregnancy body mass index, chronic hypertension, and pregestational diabetes to examine the association between the ADI [modelled in quartiles from the least (quartile 1, Q1, reference) to the most (Q4) disadvantage] and SMM. Differences in the association between ADI and SMM by self‐reported race and ethnicity as a social construct were evaluated with effect modification via an interaction term in the adjusted model.

**Main Outcomes:**

SMM, based on the US Centers for Disease Control and Prevention definition, and secondarily, SMM without transfusion.

**Results:**

Among 9588 nulliparas, 2.3% (*n* = 221) experienced any SMM and 0.5% (*n* = 48) experienced non‐transfusion SMM. Individuals living in the most disadvantaged neighbourhoods (Q4) were more likely to experience SMM compared with those in the least disadvantaged neighbourhoods (Q1) (3.4% vs. 2.1%; aRR 1.73; 95% CI: 1.17, 2.58). This association was also significant for non‐transfusion SMM (1.0% vs. 0.3%; aRR: 2.82; 95% CI 1.15, 6.93). Individuals who self‐identified as non‐Hispanic Black were more likely to experience SMM than non‐Hispanic White individuals (3.9% vs. 2.1%; *p* < 0.001). There was no evidence of effect modification by self‐reported race and ethnicity (interaction *p* > 0.05).

**Conclusion:**

Nulliparous pregnant individuals who lived in the most disadvantaged US neighbourhoods were at increased risk of experiencing SMM. Known racial and ethnic disparities in SMM may be related to adverse neighbourhood‐level social determinants.

## 1 Introduction

Every year, over 800 pregnant or postpartum individuals die in the United States (US) [1], which has the highest rate of maternal mortality (22.3 deaths per 100,000 live births) among high‐income countries [2, 3, 4]. Severe maternal morbidity (SMM), which includes unexpected complications and life‐threatening health consequences, such as organ dysfunction, end‐stage organ damage, and intensive care unit admission, is frequently on the pathway to maternal mortality [5]. The rate of SMM, which is 50 to 100 times more common than maternal mortality, has doubled in the US over the last two decades [6]. In 2021, the prevalence of SMM was 179.8 per 10,000 delivery hospitalisations [7]. Significant and persistent disparities in SMM exist between individuals from minoritised racial and ethnic groups compared with non‐Hispanic White individuals in the US; and non‐Hispanic Black individuals experience SMM at twice the rate of non‐Hispanic White individuals [7, 8].

Adverse social determinants of health (SDOH) include conditions in the environments where people are born, live, learn, work, play, worship, and age that affect health outcomes and contribute to health disparities [9]. Data increasingly highlight that individual‐level adverse SDOH, including inadequate health insurance, interpersonal racism, and lower educational attainment, are associated with an increased risk of SMM [10, 11, 12, 13, 14]. SDOH can also be measured at a broader community or neighbourhood level [15, 16]. The Area Deprivation Index (ADI) is a measure that quantifies the socioeconomic status of a neighbourhood based on US census block‐derived measures of income, education, and occupation of individuals living in the immediate geographic surroundings [15]. Such a standardised and inclusive geospatial measure of SDOH could be integrated with SMM surveillance and used to inform structural interventions, clinical practice, and public policy aimed at addressing maternal health equity [17, 18].

A relationship between the ADI and several adverse pregnancy and postpartum outcomes is well established [3, 19, 20, 21, 22, 23]. In addition, individuals from minoritised racial and ethnic subgroups are more likely to live in neighbourhoods with greater structural disadvantage (i.e., a neighbourhood with a higher ADI) [24]. Recent cross‐sectional US studies that have relied on administrative data suggest that a higher ADI is associated with an increased risk of SMM [25, 26, 27]. However, the association between the ADI early in pregnancy and consequent SMM using detailed clinical data abstraction rather than billing codes, which are prone to outcome misclassification, remains to be evaluated in a prospective, diverse, and generalisable multi‐site US cohort [28, 29].

The objective of the current analysis was to examine the association between neighbourhood socioeconomic disadvantage, as measured by the ADI in early pregnancy, and SMM at delivery hospitalisation. Because the impact of socioeconomic disadvantage associated with SMM may be greatest among racially and ethnically minoritised subgroups [30, 31], we secondarily assessed whether the above association differed by race and ethnicity.

## 2 Methods

### 2.1 Study Setting

This is a secondary analysis of the Nulliparous Pregnancy Outcomes Study: Monitoring Mothers‐to‐Be (nuMoM2b), a prospective cohort study designed to assess the influence of maternal and environmental factors on APOs [32]. Nulliparous pregnant individuals were enrolled at eight US medical centres from October 2010 to September 2013, and data were collected prospectively by standardised patient interviews, surveys, and health record abstraction at up to three time points during pregnancy and at delivery [32].

The institutional review board at each participating site approved the study, and participants provided written informed consent. Patients were not involved in the development of the research. No core outcome set was used in this research.

### 2.2 Participants

Key inclusion criteria for cohort enrolment included a singleton pregnancy with an estimated gestational age from 6 0/7 to 13 6/7 weeks, no prior delivery at 20 weeks of gestation or later, and intention to deliver at a participating hospital. Exclusion criteria included age younger than 13 years, a history of three or more pregnancy losses, donor oocyte pregnancy, planned pregnancy termination, foetal malformations likely to be lethal, known foetal aneuploidy, previous enrolment in the study, and inability to provide informed consent. For this analysis, individuals who did not report a residential address, which was required for geocoding to calculate the ADI exposure, were also excluded.

### 2.3 Exposure

The exposure was neighbourhood‐level socioeconomic disadvantage as quantified by the ADI [15]. Participants' primary residential addresses were queried at the first study visit at 6–13 weeks. This address was used to calculate the ADI. The ADI derived data from 17 US Census measures, including the domains of income, education, employment, and housing quality [15, 33]. These measures were used to quantify the socioeconomic deprivation of a geographic area, i.e., the Census Block Group [34]. The ADI is available online at https://www.neighborhoodatlast.medicine.wisc.edu.

In the current analysis, the composite score of a Census Block Group was converted to a standardised rank based on a residential locale's national percentile from 0 to 100 to allow for comparison across the US. A ranking of 1 demonstrated the least neighbourhood disadvantage, while a ranking of 100 reflected the most neighbourhood socioeconomic disadvantage. The ADI was analysed in quartiles, with the lowest quartile [Q1] representing the least socioeconomic disadvantage and serving as the reference group, and the highest quartile [Q4] representing the most socioeconomic disadvantage.

### 2.4 Outcome

The primary outcome was SMM at delivery hospitalisation. SMM without blood transfusion was secondarily assessed. SMM was categorised by a composite binary indicator (yes or no) for whether the severe maternal complications (such as eclampsia, amniotic fluid embolism, and sepsis) or life‐saving procedures (such as hysterectomy) were present. Analyses focused on a composite SMM rate, and SMM types and subtypes were not designated to be mutually exclusive. We identified SMM indicators based on the diagnoses and procedures noted in delivery hospitalisation records for each pregnancy per US Centers for Disease Control and Prevention (CDC) guidance [35]. The CDC has endorsed a surveillance algorithm that utilises diagnosis and procedure codes that indicate 21 severe life‐threatening diagnoses and procedures recorded at delivery hospitalisation discharge [6]. In the nuMoM2b cohort, trained research nurses recorded common complications (e.g., eclampsia, sepsis, need for hysterectomy) and additional clinical diagnoses or procedures (e.g., disseminated intravascular coagulation, conversion of cardiac arrhythmia). A study author (TCB) then coded these clinical diagnoses into the 21 CDC SMM categories, blinded to participant ADI. As ICD‐10 codes may overestimate blood transfusion, prior analyses frequently exclude transfusion from SMM [36]. Therefore, we conducted a secondary assessment of SMM without transfusion.

### 2.5 Covariates

Covariates were selected a priori for inclusion in multivariable analysis based on a directed acyclic graph (DAG) informed by the hypothesised relationship between SDOH and SMM (Figure S1) [37]. Models were adjusted for maternal age (years, continuous), pre‐pregnancy body mass index (BMI, kg/m^2^, continuous), chronic hypertension (yes or no), and pregestational diabetes (yes or no). Self‐reported race and ethnicity were assessed as an effect modifier in this analysis and were categorised as Asian or Pacific Islander, Black, Hispanic, White, and identification independent of these groups (American Indian or Alaskan Native, multiracial, and unreported), consistent with classifications used in US census data. Participants were classified as being Hispanic if they reported Hispanic ethnicity, regardless of race. Public or government‐financed health insurance in the US was measured by Medicaid enrollment status. Covariates were modelled as continuous measures when appropriate after assessing the underlying distribution. Imputation for missing covariate data was performed using Multiple Imputation by Chained Equations or MICE (*n* = 30 imputations), and estimates were combined using Rubin's rule.

### 2.6 Statistical Analysis

We compared the frequency of individual‐level SDOH and clinical characteristics across the exposure (ADI quartiles) using chi‐square tests for categorical variables and Wilcoxon rank sum tests for continuous variables. Modified Poisson regression was used to estimate the adjusted relative risk (aRR) and 95% confidence intervals (95% CI) between ADI quartile and SMM after adjusting for covariates. To evaluate for the presence of effect modification, an interaction term was included in the model between ADI and each minoritised race and ethnicity subgroup (Hispanic, Black, and Asian or Pacific Islander) compared with non‐Hispanic White individuals as the reference; each model to assess for interaction by subgroup was assessed separately. Imputation for missing covariate data was performed using Multiple Imputation by Chained Equations or MICE (*n* = 30 imputations), and estimates were combined using Rubin's rule. All statistical analyses were performed using STATA (StataCorp LLC, version 16.1, College Station, TX) and R statistical software version 4.2.0 (R Foundation for Statistical Computing).

## 3 Results

Among 10,038 nulliparous participants in nuMoM2b, 450 (4%) did not have first‐trimester residential address data available for geocoding. Thus, the final analytic sample included 9588 (96%) individuals. Those who did not provide a residential address were more likely to be of younger age, to self‐identify as Black or Hispanic, be Medicaid‐insured, have lower educational attainment, and have a lower household income (*p* < 0.05 for all) (Table S1). In addition, they were more likely to be living with a higher BMI, pregestational diabetes, and chronic hypertension (*p* < 0.05 for all).

The median age of the study population was 27.0 years (IQR: 22.0, 31.0) (Table 1). Twenty‐seven percent had public health insurance, 15.6% had low household income, 13.5% self‐identified as non‐Hispanic Black, and 16.7% as Hispanic. Twenty‐two percent lived with obesity (BMI ≥ 30 kg/m^2^), 1.5% with pregestational diabetes, and 2.4% with chronic hypertension.

**TABLE 1:** Socio‐demographic and clinical characteristics of nulliparous pregnant individuals overall and by Area Deprivation Index (ADI) quartile.

Variable	*N*	Overall	Quartile 1	Quartile 2	Quartile 3	Quartile 4
		*N* = 9588	*N* = 2520	*N* = 2338	*N* = 2375	*N* = 2355
ADI score, median (IQR)	9588	39.0 (18.0, 71.0)	11.0 (6.0, 15.0)	28.0 (24.0, 33.0)	53.0 (45.0, 62.0)	92.0 (84.0, 96.0)*
Age, years Median (IQR)	9588	27.0 (22.0, 31.0)	30.0 (27.0, 33.0)	28.0 (24.0, 31.0)	26.0 (22.0, 30.0) 46 (1.9)	23.0 (20.0, 27.0)*
≤ 17		228 (2.4)	13 (0.5)	38 (1.6)	2152 (90.6)	131 (5.6)
18–34		8467 (88.3)	2104 (83.5)	2092 (89.5)	157 (6.6)	2119 (90.0)
35–39		757 (7.9)	339 (13.5)	177 (7.6)	20 (0.8)	84 (3.6)
≥ 40		136 (1.4)	64 (2.5)	31 (1.3)		21 (0.9)*
Medicaid insurance	9525					
Yes		2653 (27.9)	322 (12.8)	388 (16.7)	580 (24.5)	1363 (58.7)
No		6872 (72.1)	2189 (87.2)	1941 (83.3)	1783 (75.5)	959 (41.3)*
Race and ethnicity	9588					
Non‐Hispanic White		5817 (60.7)	1769 (70.2)	1705 (72.9)	1602 (67.5)	741 (31.5)
Non‐Hispanic Black		1298 (13.5)	98 (3.9)	106 (4.5)	286 (12.0)	808 (34.3)
Hispanic		1603 (16.7)	348 (13.8)	346 (14.8)	309 (13.0)	600 (25.5)
Non‐Hispanic Asian		382 (4.0)	189 (7.5)	92 (3.9)	56 (2.4)	45 (1.9)
Other		488 (5.1)	116 (4.6)	89 (3.8)	122 (5.1)	161 (6.8)*
Education	9581					
High school or less		730 (7.6)	54 (2.1)	92 (3.9)	152 (6.4)	432 (18.4)
Some college		2965 (30.9)	377 (15.0)	620 (26.5)	812 (34.2)	1156 (49.2)
College graduate		3657 (38.2)	1052 (41.7)	1043 (44.6)	994 (41.9)	568 (24.2)
Graduate degree		2229 (23.3)	1037 (41.2)	581 (24.9)	417 (17.6)	194 (8.3)*
Tobacco use	9578					
Yes		1680 (17.5)	306 (12.2)	322 (13.8)	444 (18.7)	608 (25.9)
No		7898 (82.5)	2210 (87.8)	2015 (86.2)	1930 (81.3)	1743 (74.1)*
Body mass index, kg/m^2^	9399					
Underweight		222 (2.4)	60 (2.4)	45 (2.0)	54 (2.3)	63 (2.8)
Normal weight		4765 (50.7)	1527 (61.6)	1203 (52.1)	1112 (47.3)	923 (40.8)
Overweight		2343 (24.9)	569 (22.9)	634 (27.5)	571 (24.3)	569 (25.1)
Obesity		1131 (12.0)	212 (8.5)	248 (10.7)	342 (14.6)	329 (14.5)
Severe obesity		938 (10.0)	112 (4.5)	177 (7.7)	270 (11.5)	379 (16.7)*
Household income and size relative to the US poverty level	7846					
< 130%		5499 (70.1)	2057 (89.6)	1580 (78.4)	1310 (66.5)	552 (35.2)
130%–350%		1126 (14.4)	134 (5.8)	261 (13.0)	362 (18.4)	369 (23.6)
> 350%		1221 (15.6)	104 (4.5)	174 (8.6)	298 (15.1)	645 (41.2)*
Pregestational diabetes	9155					
Yes		137 (1.5)	16 (0.7)	28 (1.2)	35 (1.5)	58 (2.6)
No		9018 (98.5)	2381 (99.3)	2213 (98.8)	2248 (98.5)	2176 (97.4)*
Chronic hypertension	9063					
Yes		217 (2.4)	37 (1.6)	50 (2.3)	49 (2.2)	81 (3.7)
No		8846 (97.6)	2342 (98.4)	2164 (97.7)	2213 (97.8)	2127 (96.3)*

*Note:* Chi‐square test was used to compare categorical variables and Wilcoxon rank sum test for continuous variables.

* *p* < 0.05 for assessed characteristics.

The median ADI score was 39.0 (interquartile range [IQR]: 18.0, 71.0) (Table 1). The median ADI score by quartile was 11.0 (IQR: 6.0, 15.0) for Q1, 28.0 (IQR: 24.0, 33.0) for Q2, 53.0 (IQR: 45.0, 62.0) for Q3 and 92.0 (IQR: 84.0, 96.0) for Q4. Individuals living in a neighbourhood with a higher ADI quartile were more likely to self‐identify as Black or Hispanic, have public health insurance, have lower educational attainment, and have a lower household income (*p* < 0.05 for all). They were also more likely to live with a higher BMI, pregestational diabetes, and chronic hypertension (*p* < 0.05 for all).

**FIGURE 1:**
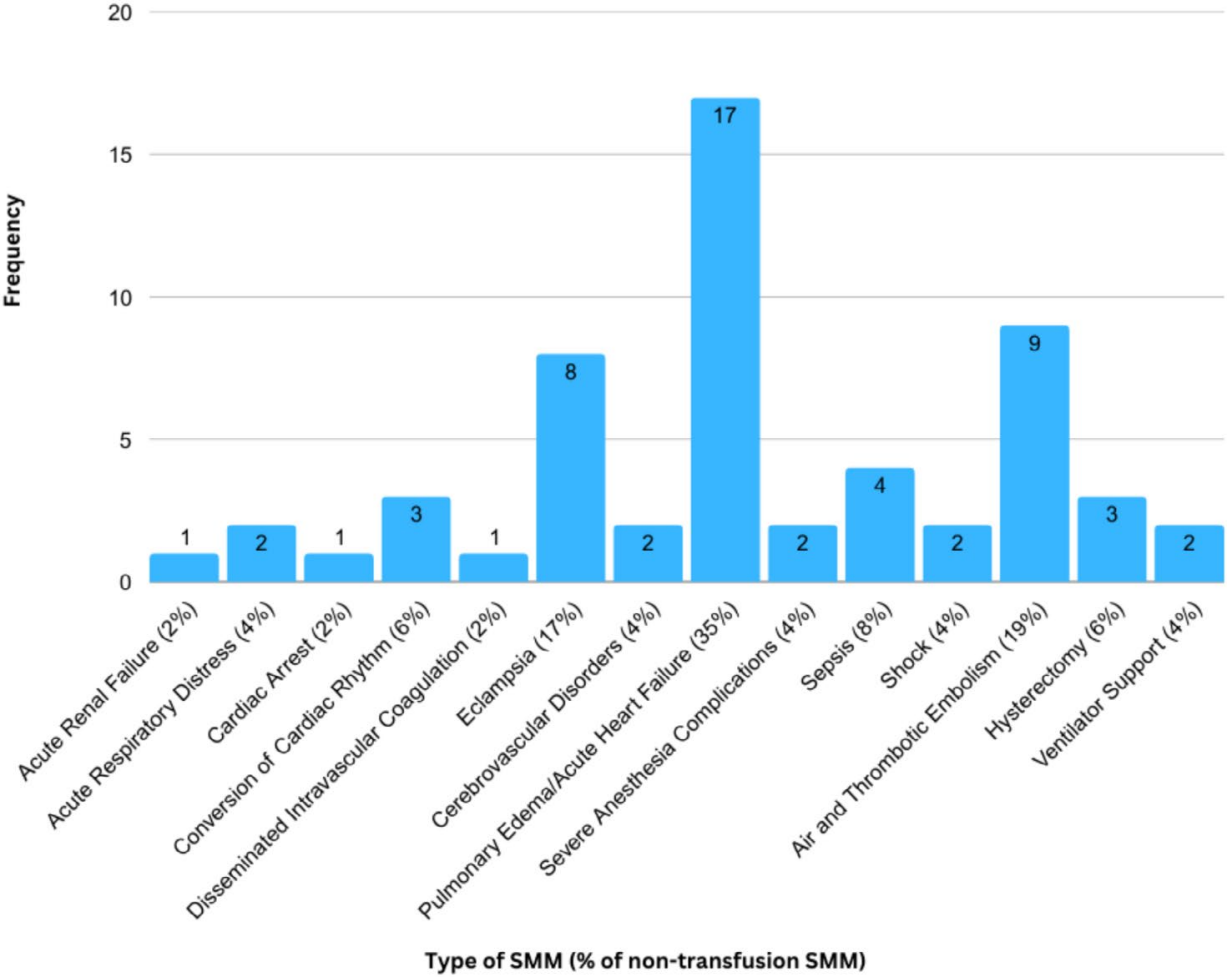
Frequency of non‐transfusion SMM.

**FIGURE 2:**
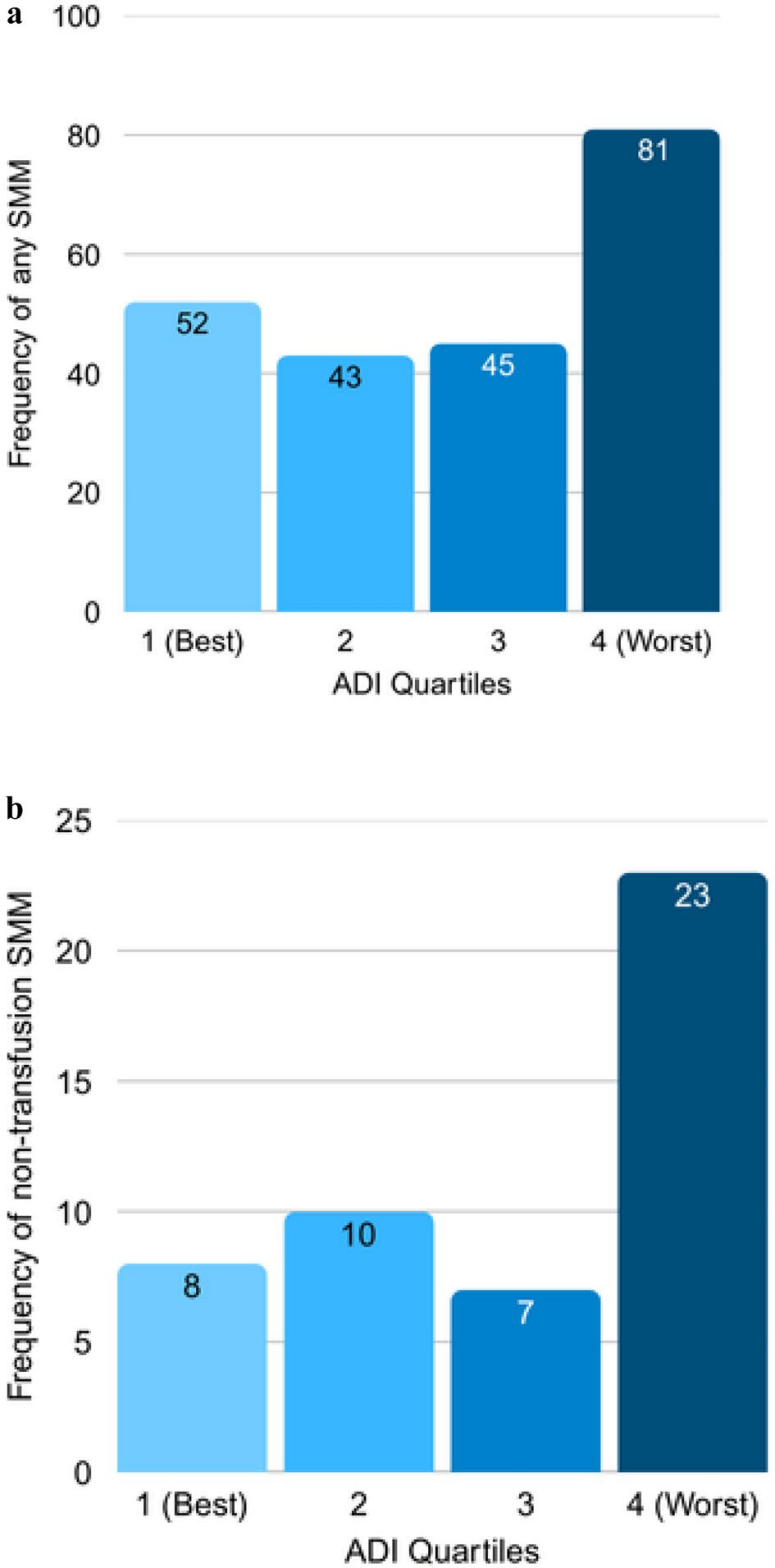
(a) Frequency of any SMM by ADI quartile. (b) Frequency of non‐transfusion SMM by ADI quartile.

Overall, 2.3% (*n* = 221) of the study population experienced any SMM and 0.5% (*n* = 48) experienced non‐transfusion SMM (Figure 1). Individuals who self‐identified as non‐Hispanic Black were more likely to experience SMM than non‐Hispanic White individuals (3.9% vs. 2.1%, *p* < 0.001). Individuals living in the most disadvantaged neighbourhoods (Q4) were more likely to experience SMM compared with those in the least disadvantaged neighbourhoods (Q1) (3.4% vs. 2.1%; aRR 1.73; 95% CI: 1.17, 2.58) (Table 2, Figure 2a). In secondary analysis, living in the most disadvantaged neighbourhoods was also significantly associated with non‐transfusion SMM (1.0% vs. 0.3%; aRR 2.82; 95% CI: 1.15, 6.93) (Figure 2b).

**TABLE 2:** Association of neighbourhood‐level socioeconomic disadvantage in early pregnancy and severe maternal morbidity (SMM) at delivery hospitalisation.

	*N*	Frequency of SMM (row percentage)		Unadjusted risk ratio^a^, RR (95% CI)	Adjusted risk ratio^a^, ^b^, aRR (95% CI)
		No *n* (%)	Yes *n* (%)		
**Any SMM**					
**Area Deprivation Index**					
Quartile 1 (least deprived)	2520	2468 (97.9)	52 (2.1)	1.00	1.00
Quartile 2	2338	2295 (98.2)	43 (1.8)	0.89 (0.60, 1.33)	0.91 (0.60, 1.37)
Quartile 3	2375	2330 (98.1)	45 (1.9)	0.92 (0.62, 1.36)	0.94 (0.62, 1.42)
Quartile 4 (most deprived)	2355	2274 (96.6)	81 (3.4)	**1.67 (1.18, 2.35)**	**1.73 (1.17, 2.58)**
**Non‐transfusion SMM**					
**Area Deprivation Index**					
Quartile 1 (least deprived)	2520	2512 (99.7)	8 (0.3)	1.00	1.00
Quartile 2	2338	2328 (99.6)	10 (0.4)	1.35 (0.53, 3.41)	1.33 (0.52, 3.40)
Quartile 3	2375	2368 (99.7)	7 (0.3)	0.93 (0.34, 2.56)	0.88 (0.31, 2.48)
Quartile 4 (most deprived)	2355	2332 (99.0)	23 (1.0)	**3.08 (1.38, 6.86)**	**2.82 (1.15, 6.93)**

*Note:* Bolded results signify statistically significant findings (*p* < 0.05).

^a^ Poisson regression with robust error variance with imputation for missing covariates was used to estimate relative risk (RR) and adjusted relative risk (aRR).

^b^ Adjusted model included individual‐level covariates: age, pre‐pregnancy body mass index, chronic hypertension, and pregestational diabetes.

Interaction effects (effect modification) between self‐reported race and ethnicity and the main exposure of ADI were not significant in the above adjusted models (*p* = 0.85 for non‐Hispanic Black, 0.55 for Hispanic, and 0.27 for non‐Hispanic Asian or Pacific Islander subgroups compared with non‐Hispanic White as the reference). Hence, additional stratified analyses were not performed.

## 4 Discussion

### 4.1 Main Findings

In a prospective multi‐site cohort, nulliparous pregnant individuals who lived in the most disadvantaged US neighbourhoods were at increased risk of experiencing SMM. In interaction analyses, these results were similar regardless of minoritised racial and ethnic status, suggesting that residence in structurally disadvantaged neighbourhoods, more frequent among minoritised populations, may contribute to racial and ethnic disparities in SMM.

### 4.2 Strengths and Limitations

Strengths of this study include the use of the ADI, which is publicly accessible to researchers and clinicians through a web‐based portal. Compared with prior studies focused on SMM that have primarily relied on ICD‐10 codes, the current analysis employed diagnoses and procedures for defining SMM, which may more accurately capture clinical conditions and severity and be less prone to misclassification [28]. Using ICD‐10 criteria without further clinical validation leads to a high rate of misclassification, with a positive predictive value of less than 50% [28, 29]. The current analysis measured the ADI in early pregnancy, as opposed to other cross‐sectional analyses, which measured the ADI at the time of delivery. Finally, these data were collected in a prospective, longitudinal approach and included a geographically diverse cohort of pregnant individuals from across the US, increasing the generalisability of results.

There are several limitations to note. First, the study excluded individuals for whom an ADI could not be calculated; such individuals may be more likely to experience unstable housing and therefore would have a higher ADI and be at greater risk for SMM [38]. Hence, excluding such individuals may have biased our results towards the null. Second, this study was restricted to nulliparous individuals who initiated prenatal care in the first trimester and chose to participate in a prospective cohort. It is possible that individuals at higher risk of SMM may have been less likely to enrol in prenatal care in the first trimester [39]. However, the rate of SMM in the current study is consistent with prior data [7]. While these data were also collected between 2010 and 2013, the rate of SMM has increased and disparities in maternal outcomes attributed to adverse SDOH persist [40]. Hence, it is unlikely that the observed association between ADI and SMM would be different with more recent data, and it is even possible that the association was underestimated using older data. Finally, the relationship between SDOH including minoritised race and ethnicity and socioeconomic disadvantage is complex and varies across regional settings, and in the current study, such analyses were exploratory.

### 4.3 Interpretation

These findings extend what is known about the relationship between neighbourhood‐level SDOH and adverse pregnancy outcomes [e.g., hypertensive disorders of pregnancy [21, 41], gestational diabetes mellitus [22], stillbirth [23], and maternal Group B *streptococcal* colonisation [42]] and postpartum outcomes [e.g., cardiovascular disease risk [43] and readmission [20]]. A cross‐sectional study using linked Michigan statewide birth records and Medicaid claims data identified an association between ADI at delivery and SMM [25]. Similarly, two cross‐sectional analyses using delivery admissions within single healthcare systems also found an association between ADI and SMM [26, 27]. Prior analyses also examined specific indicators of neighbourhood‐level adversity associated with SMM, such as racial and economic spatial polarisation, violent crimes, housing violations, renter‐occupied housing units, housing violations, and receipt of public assistance [10, 11, 13]. While Guglielminotti et al. found that multiple neighbourhood deprivation markers were associated with SMM in unadjusted analyses, these associations were no longer significant in adjusted analyses that accounted for individual‐level factors [12].

In the current analysis, the association between ADI and SMM was similar across racial and ethnic subgroups. However, minoritised individuals were more likely to live in neighbourhoods with greater or worse ADI. This may reflect the impact of structural racism through racial residential segregation [44, 45]. Prior data suggest that neighbourhood deprivation, including racialised economic segregation, is associated with SMM among non‐Hispanic Black and Hispanic individuals [11, 25, 26, 46].

There are several mechanisms through which adverse neighbourhood conditions may lead to SMM. It has been hypothesised that adverse SDOH may lead to poor pregnancy outcomes by influencing an individual's interaction with the healthcare system, limiting access to healthy food and exercise options, and increasing chronic stress [3, 21, 22]. Neighbourhood residence also affects the location of delivery and the availability of hospital resources. Existing data demonstrate the effect of hospital‐level SDOH and the risk of SMM [12, 47]. Future work exploring the interaction between hospital‐level factors and patient behaviours, including healthcare utilisation, is needed to understand the impact of these variables on the association between adverse neighbourhood‐level SDOH and maternal health. Data from outside pregnancy suggest that higher ADI is associated with increased healthcare spending and patient‐reported barriers to care due to cost [48].

## 5 Conclusions

In a prospective, geographically diverse, US cohort, living in a neighbourhood with greater social disadvantage was associated with an increased risk of SMM at delivery hospitalisation at the time of first livebirth. Understanding the influence of neighbourhood‐level SDOH on SMM can inform future community‐based care delivery, multi‐level structural interventions, and public policies that prioritise investment in disadvantaged neighbourhoods to improve maternal health equity.

## Author Contributions

T.C.B., W.A.G., and K.K.V. contributed to initial study design and primary authorship of the manuscript. All authors contributed to data interpretation, manuscript development, and revision. J.W. provided primary statistical support for this manuscript.

## Conflicts of Interest

The authors declare no conflicts of interest.

## Supporting information


**Data S1:** bjo70024‐sup‐0001‐DataS1.docx.

## Data Availability

The data that support the findings of this study are available from the corresponding author upon reasonable request.
