# Supplementary material for: Neighbourhood Socioeconomic Disadvantage and Severe Maternal Morbidity: Secondary Analysis of a Prospective Cohort
Source: BJOG. 2025 Sep 30;132(13):2256–64. doi: 10.1111/1471-0528.70024 (PMC12592760; doi:10.1111/1471-0528.70024)
Supplement: Supplementary file 1 — Data S1: bjo70024‐sup‐0001‐DataS1.docx. [file BJO-132-2256-s001.docx]

| **Supplementary Table 1. Socio-demographic and clinical characteristics between overall sample and final analytic sample** | | | |
| --- | --- | --- | --- |
| **Variable** | **Overall**  **N = 10,038** | **Excluded**  **N=450** | **Included^1^**  **N=9,588** |
|  | **N (%)** | **N (%)** | **N (%)** |
| Age, years, median (IQR) (N=10,028)  <17  18-34  35-39  ≥40 | 27.0 (22.0, 31.0)  244 (2.4)  8,852 (88.3)  788 (7.9)  144 (1.4) | 25.0 (20.0, 30.0)  16 (3.6)  385 (87.5)  31 (7.0)  8 (1.8) | 27.0 (22.0, 31.0)*  228 (2.4)  8,467 (88.3)  757 (7.9)  136 (1.4) |
| Medicaid insurance (N=9,959)  Yes  No | 2,854 (28.7)  7,105 (71.3) | 201 (46.3)  233 (53.7) | 2,653 (27.9)  6,872 (72.1)* |
| Race and ethnicity (N=10,028)  Non-Hispanic White  Non-Hispanic Black  Hispanic  Non-Hispanic Asian  Other | 5,989 (59.7)  1,418 (14.1)  1,700 (17.0)  407 (4.1)  514 (5.1) | 172 (39.1)  120 (27.3)  97 (22.0)  25 (5.7)  26 (5.9) | 5,817 (60.7)  1,298 (13.5)  1,603 (16.7)  382 (4.0)  488 (5.1)* |
| Education (N=10,020)  High school or less  Some college  College graduate  Graduate degree | 816 (8.1)  3,119 (31.1)  3,777 (37.7)  2,308 (23.0) | 86 (19.6)  154 (35.1)  120 (27.3)  79 (18.0) | 730 (7.6)  2,965 (30.9)  3,657 (38.2)  2,229 (23.3)* |
| Tobacco use (N=10,018)  Yes  No | 1,782 (17.8)  8,236 (82.2) | 102 (23.2)  338 (76.8) | 1,680 (17.5)  7,898 (82.5)* |
| Body mass index, kg/m^2^ (N=9,812)  Underweight  Normal weight  Overweight  Obese  Severely obese | 230 (2.3)  4,966 (50.6)  2,444 (24.9)  1,170 (11.9)  1,002 (10.2) | 8 (1.9)  201 (48.7)  101 (24.5)  39 (9.4)  64 (15.5) | 222 (2.4)  4,765 (50.7)  2,343 (24.9)  1,131 (12.0)  938 (10.0)* |
| Household income and size relative to the U.S. poverty level (N=8,128)  <130%  130 to 350%  >350% | 5,662 (69.7)  1,169 (14.4)  1,297 (16.0) | 163 (57.8)  43 (15.2)  76 (27.0) | 5,499 (70.1)  1,126 (14.4)  1,221 (15.6)* |
| Pregestational diabetes (N=9,566)  Yes  No | 151 (1.6)  9,415 (98.4) | 14 (3.4)  397 (96.6) | 137 (1.5)  9,018 (98.5)* |
| Chronic hypertension (N=9,469)  Yes  No | 243 (2.6)  9,226 (97.4) | 26 (6.4)  380 (93.6) | 217 (2.4)  8,846 (97.6)* |
| ^1^Chi-square test was used to compare categorical variables and Wilcoxon rank sum test for continuous variables. p<0.05 for all assessed characteristics above. | | | |

**Supplementary Figure 1. Directed Acyclic Graph.**
